# Deubiquitinating enzyme USP37 regulating oncogenic function of 14-3-3γ

**DOI:** 10.18632/oncotarget.5336

**Published:** 2015-09-25

**Authors:** Jin-Ock Kim, So-Ra Kim, Key-Hwan Lim, Jun-Hyun Kim, Brijesh Ajjappala, Hey-Jin Lee, Jee-In Choi, Kwang-Hyun Baek

**Affiliations:** ^1^ Department of Biomedical Science, CHA University, Bundang CHA Hospital, Gyeonggi-Do 463-400, Republic of Korea; ^2^ Department of Rehabilitation Medicine, CHA University, Bundang CHA Hospital, Gyeonggi-Do 463-400, Republic of Korea

**Keywords:** 14-3-3, cell proliferation, deubiquitinating enzyme, ubiquitin-specific protease

## Abstract

14-3-3 is a family of highly conserved protein that is involved in a number of cellular processes. In this study, we identified that the high expression of 14-3-3γ in various cancer cell lines correlates with the invasiveness of the cancer cells. Overexpression of 14-3-3γ causes changes to the morphologic characteristics of cell transformation, and promotes cell migration and invasion. The cells overexpressed with 14-3-3γ have been shown to stimulate foci and tumor formation in SCID-NOD mice in concert with signaling components as reported with the 14-3-3β. In our previous study, we demonstrated that 14-3-3γ inhibits apoptotic cell death and mediates the promotion of cell proliferation in immune cell lines. Earlier, binding partners for 14-3-3γ were defined by screening. We found that USP37, one of deubiquitinating enzymes (DUBs), belongs to this binding partner group. Therefore, we investigated whether 14-3-3γ mediates proliferation in cancer cells, and 14-3-3γ by USP37 is responsible for promoting cell proliferation. Importantly, we found that USP37 regulates the stability of ubiquitin-conjugated 14-3-3γ through its catalytic activity. This result implies that the interactive behavior between USP37 and 14-3-3γ could be involved in the regulation of 14-3-3γ degradation. When all these findings are considered together, USP37 is shown to be a specific DUB that prevents 14-3-3γ degradation, which may contribute to malignant transformation via MAPK signaling pathway, possibly providing a new target for therapeutic objectives of cancer.

## INTRODUCTION

Ubiquitination of proteins through the ubiquitin-proteasome pathway (UPP) is an important post-translational modification (PTM) involved in regulating the levels of most cellular proteins and eliminating misfolded proteins. PTM by ubiquitin plays an important role in a variety of cellular functions [1–3]. Protein ubiquitination is mediated by sequential enzymatic actions via ubiquitin-activating enzymes (E1), ubiquitin-conjugating enzymes (E2), and ubiquitin ligases (E3). An ubiquitination factor, (E4), which is required for efficient polyubiquitination, has been identified in yeast [4, 5]. Throughout these processes, targeted proteins are directed toward ATP-dependent hydrolysis by the 26S proteasome [6]. Deubiquitination, the reversal process of ubiquitination, is catalyzed by deubiquitinating enzymes (DUBs), which remove ubiquitin from conjugated target proteins [1, 5, 7]. The human genome codes for approximately 100 DUBs. Most DUBs are cysteine proteases, and they are classified into at least six families [8, 9].

A broad range of organisms and tissues contain 14-3-3 proteins. They have many diverse functions, including central roles in the signal transduction pathway, exocytosis, anti-apoptotic process, and cell cycle regulation. Many signaling pathways involving 14-3-3 proteins are overactivated during tumorigenesis, indicating that these proteins can bind to a number of target proteins altered in various types of cancer involving alterations of YSK1, TAZ, integrin α, and ErbB2 [10–13 ]. Among 14-3-3 proteins, 14-3-3γ has been shown to induce oncogenic transformation. In contrast, 14-3-3σ was found to act as a tumor suppressor protein [14, 15]. Mitogen-activated protein kinase (MAPK) signaling is known to play a critical role in various cancers, stimulating the growth of cancer cells. Oncogenic transformation associated with MAPK has been shown to be linked to the function of 14-3-3γ [15]. We previously demonstrated that murine 14-3-3γ can trigger oncogenesis, promoting cell proliferation in leukemic cell lines [16], and Kasahara *et al*. reported that 14-3-3γ-mediated growth signaling can induce the metaphase-anaphase transition by regulating phosphorylation of a specific polo-like kinase 1 (Plk1) serine residue, which is regarded as a mitosis-specific phosphorylation site [17].

Based on our previous finding that 14-3-3γ seemed to be involved in cell proliferation [16] and that ubiquitin-specific protease 37 (USP37) was one of the binding partners of 14-3-3γ [18], we investigated the function of 14-3-3γ in tumorigenesis and confirmed the interaction between USP37 and 14-3-3γ. We evaluated the interaction of USP37 with 14-3-3γ by co-immunoprecipitation (co-IP) and glutathione S-transferase (GST) pull-down assays. We demonstrated that 14-3-3γ underwent polyubiquitination and that USP37 acted as a specific DUB for 14-3-3γ. Based on our experimental results, USP37 appears to play an important role in cell proliferation in cancer, regulating the stability of 14-3-3γ. Taken together, the results suggest that USP37 can be considered a therapeutic target for the regulation of cell proliferation and a biomarker of tumorigenesis in cancers.

## RESULTS

### Overexpression of 14-3-3γ induces tumorigenic transformation of Ba/F3 cells

**Figure 1:**
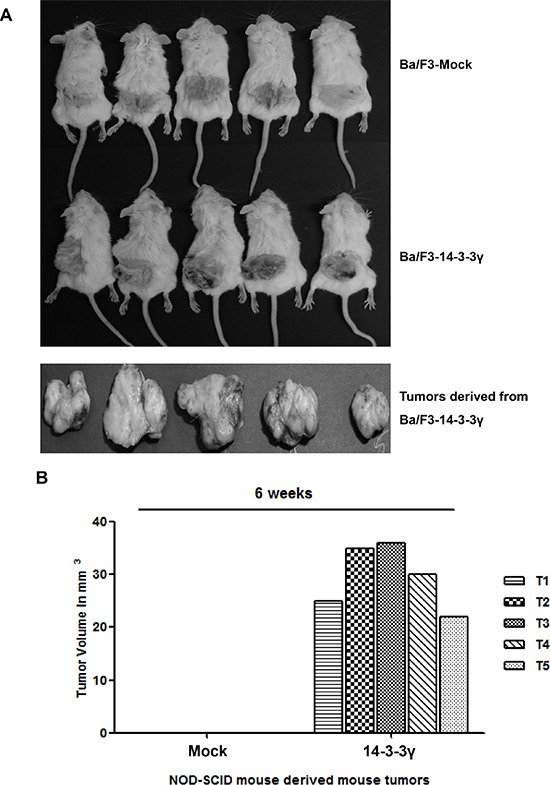

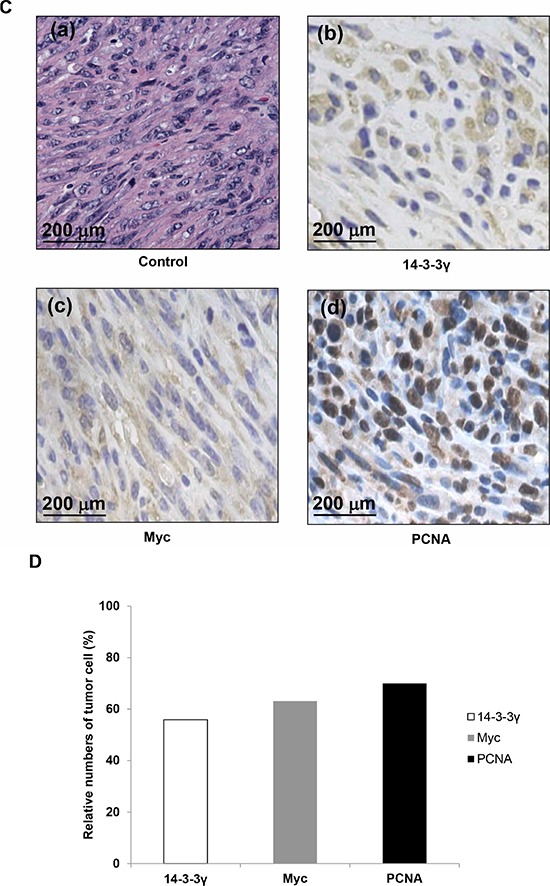
Tumorigenicity of 14-3-3γ **A.** Ba/F3 cells (2 × 10^6^) stably transfected with either vector were injected subcutaneously into SCID-NOD mice. *n* = 5. **B.** The tumor size after 6 weeks ranged from 25 to 36 mm^3^. **C.** Immunohistochemical analysis of 14-3-3γ-derived mouse tumors. Ba/F3–14-3-3γ tumor cells stained with hematoxylin and eosin showing a negative control (a) and antibodies specific for 14-3-3γ (b), c-Myc (c), and PCNA (d). Scale bar = 200 μm. **D.** Percentage of 14-3-3γ-, Myc-, and PCNA-expressing tumor cells, respectively.

Previously, we revealed that overexpression of 14-3-3γ increased cell proliferation through activation of the phosphoinositide 3-kinase (PI3K) and MAPK signaling cascades in the absence of interleukin-3 (IL-3) stimulation [16]. This result led us to investigate the molecular regulation of 14-3-3γ in cancer cells. In addition, based on the ability of anchorage-independent growth *in vitro* and the focus-forming ability of NIH3T3 cells with the overexpression of 14-3-3γ under reduced serum conditions, we first investigated the effect on tumorigenesis of the *in vivo* growth characteristics using 14-3-3γ overexpressed Ba/F3 cells. In that study, we subcutaneously transplanted Ba/F3 cells into the flanks of non-obese diabetic/severe combined immunodeficiency (NOD/SCID) mice, which were transfected with either an empty vector or 14-3-3γ. In each experiment, a group of five mice was used. The results showed that Ba/F3 cells expressing 14-3-3γ induced tumors and that these tumors grew rapidly (Figure 1A). The mice transplanted with the mock-transfected cells did not develop tumors even after 80 days. All the tumor-bearing mice were sacrificed 6 weeks after transplantation, and the tumor volumes were determined. The average volume of the tumors was 30 mm^3^ (Figure 1B). Gross examination of the organs revealed no metastatic spread to other organs, but this was likely due to the short 6-week study period.

The tumors generated by the Ba/F3 cells overexpressing 14-3-3γ were excised and analyzed by immunohistochemistry to determine the expression of c-Myc, on account of its cooperative action on tumor growth with 14-3-3γ. Proliferating cell nuclear antigen (PCNA), which act as a sensor molecule, is regulated by 14-3-3 during DNA damage [19]. In this study, more than 50% of the tumor cells were positive for nuclear expression of 14-3-3γ, Myc, and PCNA (Figure 1C and 1D). The morphological features of all the tumors were similar. The tumors showed high cellularity, which consisted of spindle cells, some with atypical nuclei and forming fascicles highly suggestive of a fibrosarcoma. These results demonstrated that the overexpression of 14-3-3γ rendered Ba/F3 cells tumorigenic *in vivo*.

### 14-3-3γ promotes cancer cell growth

**Figure 2:**
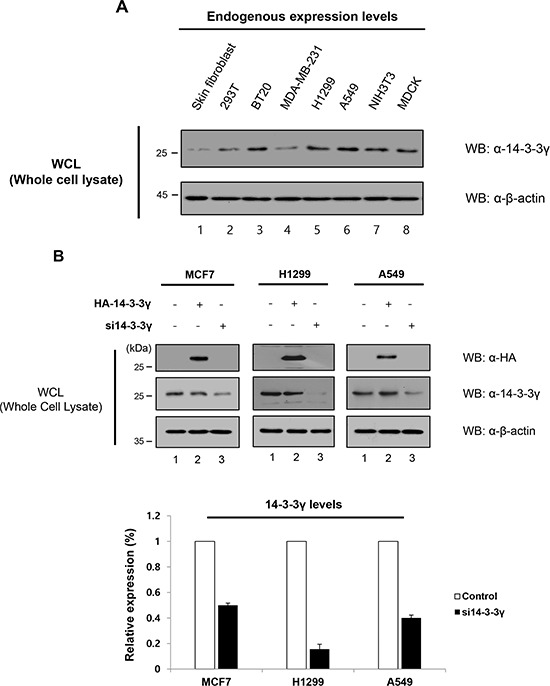

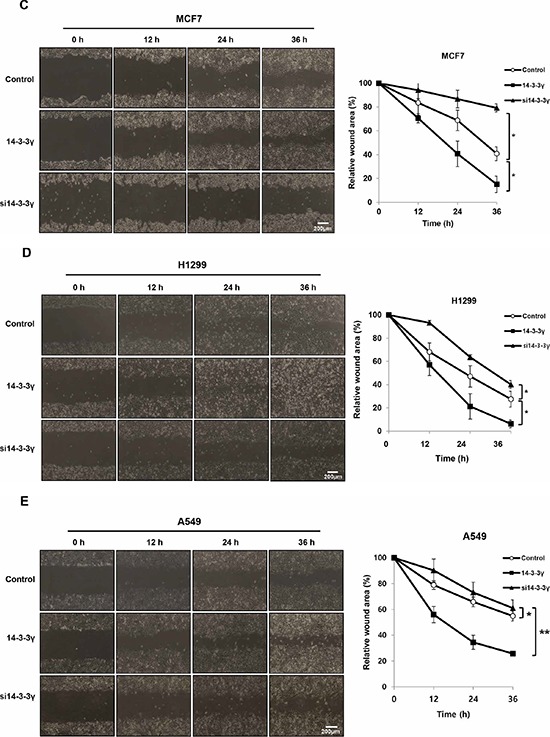

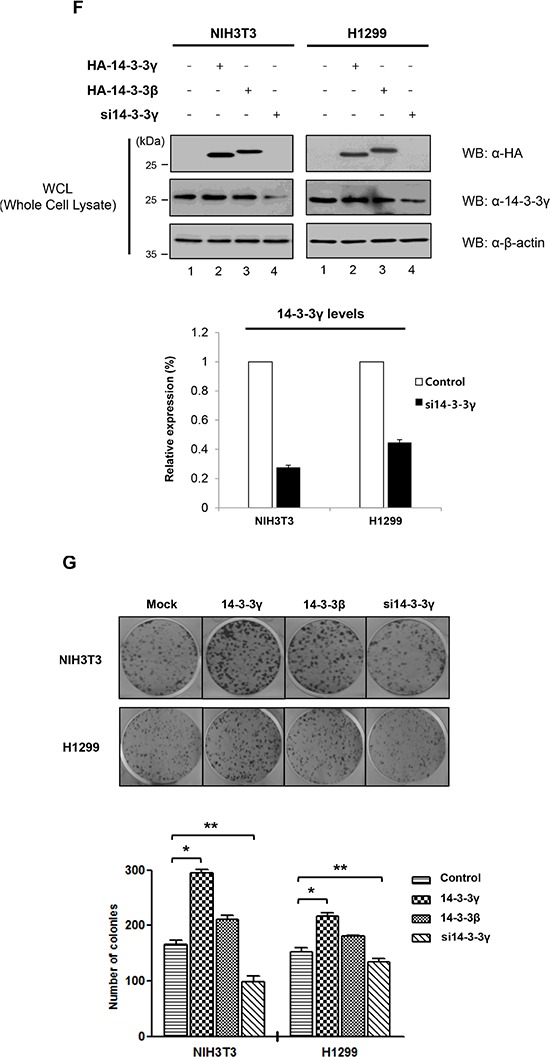
Promotion of cell proliferation by overexpression of 14-3-3γ in cancer cells **A.** The expression of endogenous 14-3-3γ was investigated in various cancer cells, including breast and lung, in addition to immortalized cancerous cell lines and, normal cells. Cell lysates were used for immunoblotting with an anti-14-3-3γ antibody. **B.** 14-3-3γ-mediated migratory and invasive potential in MCF7, H1299, and A549 cells were investigated in a wound-healing assay. *n* = 3. **C, D, and E.** Wound healing by migrated cells at 0, 12, 24 and 36 h was imaged. Scale bar = 200 μm. The percentage of migration was statistically analyzed from separate experiments and graphed using Graph Pad Prism Software. The data are presented as means ± s.d. (Student’ *t*-test) *P < 0.01, *n* = 3. **F.** NIH3T3 and H1299 cells were transfected with HA-*14-3-3γ* and HA-*14-3-3β*. Additionally, siRNA specific for 14-3-3γ was used to investigate the effect of knock-down of endogenous 14-3-3γ. *n* = 3. H, Colony formation assay. NIH3T3 and H1299 cells stably expressing an empty vector, HA-14-3-3γ, HA-14-3-3β, and *si14-3-3γ* were plated in triplicate. *n* = 3. **G.** After 14 days, the colonies were stained and counted. *n* = 3. The number of colonies formed was graphed using Graph Pad Prism Software. The results represent the average number of colonies formed from three independent experiments. The data are presented as means ± s.d. *P < 0.01 and **P < 0.05, *n* = 3.

In our previous study, we found that 14-3-3γ was upregulated in a murine pro-B cell line (Ba/F3) by IL-3 stimulation and that overexpression of 14-3-3γ resulted in IL-3-independent proliferation of Ba/F3 cells [16]. A previous study reported that 14-3-3 proteins were overexpressed in various cancers, including lung, breast, ovarian, and colorectal cancers, suggesting that 14-3-3γ might play a role in tumorigenesis [20, 21]. Therefore, we analyzed the expression level of 14-3-3γ in various cancer cells and normal cells (Figure 2A). The results revealed that the expression of 14-3-3γ was higher in cancer cells than in normal cells, indicating that 14-3-3γ might be involved in cancer.

To examine the molecular functions of 14-3-3γ in cancer cell proliferation, we overexpressed or knocked-down 14-3-3γ in breast and lung cancer cells (Figure 2B). After checking the relative expression levels of 14-3-3γ, we performed a cell-based assay to evaluate cell migration. Due to cell migration of the 14-3-3γ overexpressed cells, the wound area recovered more rapidly (within 36 h) compared to the recovery in the control. However, in the same period, the wound closure was delayed in the 14-3-3γ knockdown group (Figure 2C–2E). Interestingly, cell proliferation was the slowest in the group in which 14-3-3γ was depleted (Figure 2C–2E). We observed that closure of the wound area in human non-small cell lung carcinoma cell line, H1299, was rapid compared with that in breast cancer cell line, MCF7, during the same period, although knocking down 14-3-3γ had a greater effect on the H1299 cells than on the MCF7 cells (Figure 2B–2D). An identical experiment was also performed in human lung carcinoma cell line, A549, and we identified a similar effect (Figure 2E). As is known, the doubling times of various types of cells, including cancer cells, differ. These results suggest that the doubling times of cancer cells influence cell proliferation. Overall, our results indicate that the expression of 14-3-3γ accelerates cancer cell proliferation.

It was previously shown that the overexpression of 14-3-3β mediated cell growth and induced tumorigenicity via the activation of the MAPK cascade [22]. In parallel, enforced expression of antisense 14-3-3β inhibited cell proliferation, tumorigenicity, and angiogenesis. To determine the effect of 14-3-3γ versus that of 14-3-3β, we overexpressed both 14-3-3γ and 14-3-3β in NIH3T3 cells (Figure 2F). The number of colonies induced by the 14-3-3γ overexpression was increased 1.8-fold, whereas those induced by the 14-3-3γ-knocked-down cells decreased 1.4-fold compared with those of the controls (Figure 2G). Interestingly, the colony formation of the 14-3-3γ-overexpressed cells was also higher than that of the 14-3-3β-overexpressed cells. To exclude the effect of the cell line of origin, the level of colony formation induced by 14-3-3γ overexpression was assessed in H1299 p53-null cells (Figure 2F and 2G). The results showed that the overexpression of 14-3-3γ also affected colony formation in the H1299 cells (Figure 2F and 2G). Previous studies revealed that 14-3-3γ-overexpressed cells had a strong stimulatory effect on growth properties, such as growth rates, saturation density, and foci-forming ability. The ability of 14-3-3γ overexpression to promote colony formation suggests that it might also play a role in cell transformation, as shown in studies of 14-3-3β, via the MAPK signaling pathway [15, 23]. Consistently, these results demonstrate that the expression of 14-3-3γ is strongly associated with cancer cell growth, activating the MAPK signaling pathway.

### USP37 is one of the binding partners of 14-3-3γ

**Figure 3:**
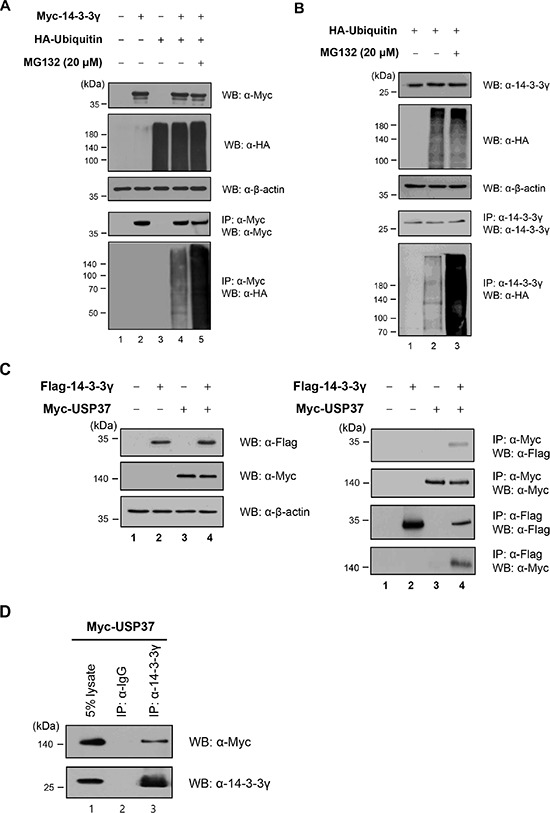

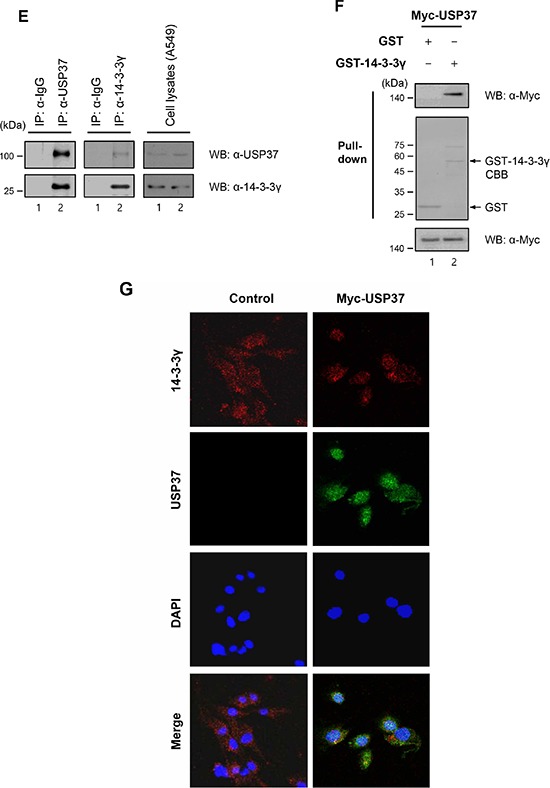

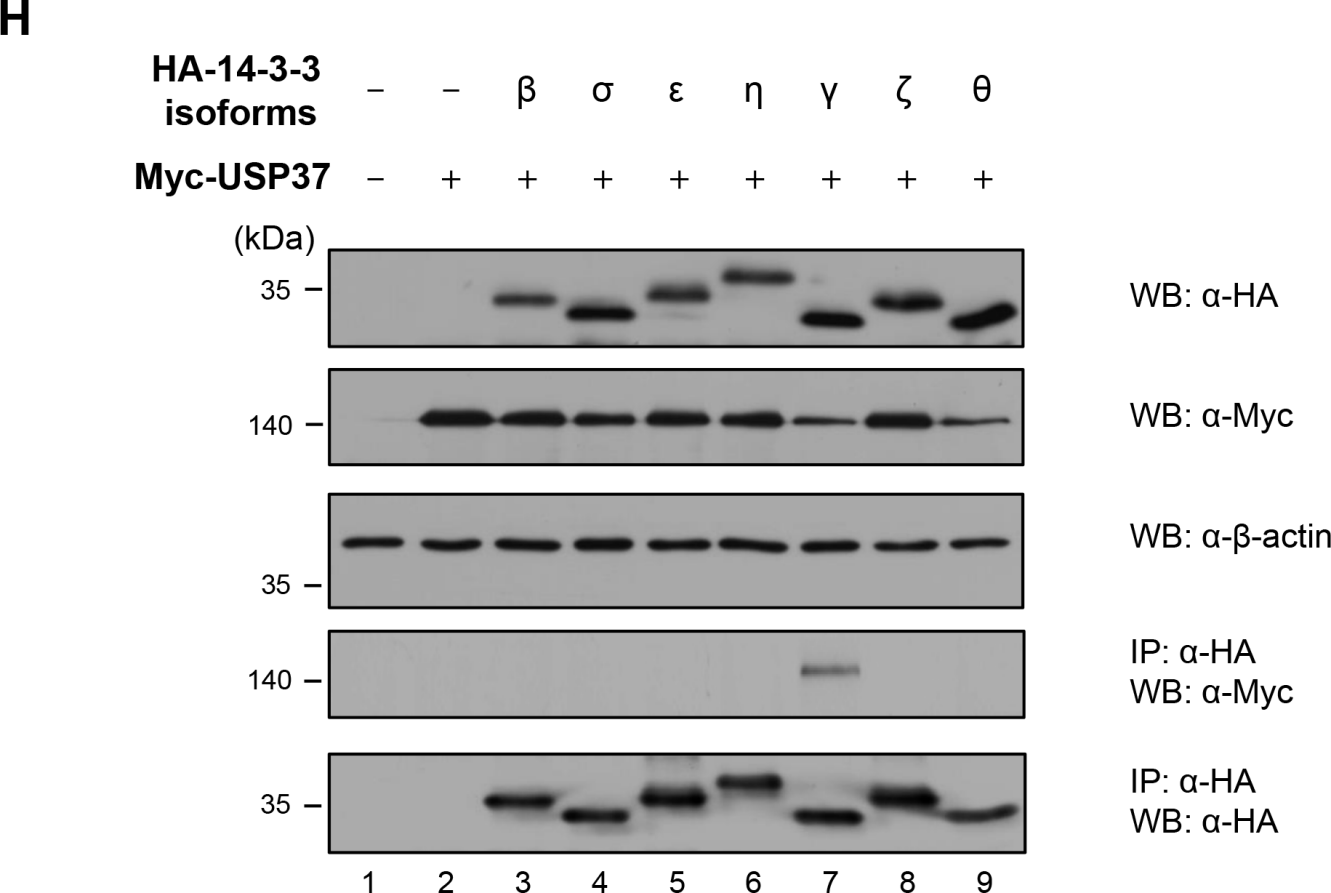
14-3-3γ undergoes polyubiquitination and directly binds to USP37 **A.** 293T cells were transfected with Myc-*14-3-3γ* and HA-*ubiquitin* separately and together. These cells were harvested after treatment with MG132 for 4 h. Ubiquitination of 14-3-3γ was confirmed by co-IP with an anti-Myc antibody and immunoblotting with anti-HA and anti-Myc antibodies. **B.** Cell lysates expressing HA-Ubiquitin treated with MG132 were immunoprecipitated with an anti-14-3-3γ antibody. **C.** *In vivo* binding assay of USP37 and 14-3-3γ. 293T cells were transfected with Myc-*Usp37* and/or Flag-*14-3-3γ*. The interaction between USP37 and 14-3-3γ was confirmed by co-IP with either an anti-Myc antibody and immunoblotting with an anti-Flag antibody. **D.** Myc-USP37 overexpressing 293T cell lysates were used for IP with an anti-14-3-3γ antibody. **E.** A549 cell lysates were used in an IP assay to examine the interaction between endogenous USP37 and 14-3-3γ. **F.** GST-14-3-3γ-bound beads were incubated with USP37 overexpressing cell lysate. Bound USP37 proteins were immunoblotted with an anti-Flag antibody (upper panel) and stained with Coomassie Brilliant Blue (middle panel). **G.** NIH3T3 cells were incubated with anti-14-3-3γ and anti-Myc antibodies followed by FITC staining. DAPI was used to counterstain the nucleus. (green: Myc-USP37, red: 14-3-3γ, blue: DAPI, and yellow: co-localization). **H.** For the binding assay between USP37 and other 14-3-3 isoforms, HA-*14-3-3* isoforms were transfected into 293T cells, together with Myc-*USP37*.

Our previous results showed that the expression level of 14-3-3γ regulated cell proliferation [16] (Figure 2). Subsequently, we focused on the ubiquitination of 14-3-3γ as a PTM. As most proteins are degraded within the 26S proteasome, we assessed the conjugation of 14-3-3γ with ubiquitin molecules. To date, no studies have examined the ubiquitination of 14-3-3γ. We tested whether 14-3-3γ underwent ubiquitination in the cells exogenously overexpressing 14-3-3γ and ubiquitin. The results revealed that MG132, a proteasome inhibitor, affected the accumulation of 14-3-3γ ubiquitination (Figure 3A). In addition, we analyzed the ubiquitination of endogenous 14-3-3γ (Figure 3B). Using an anti-HA antibody, Western blot analysis showed that a 14-3-3γ antibody precipitated ubiquitin molecules conjugated with 14-3-3γ and that MG132 increased the ubiquitination level of 14-3-3γ (Figure 3B, lanes 2 and 3). Thus, our results indicated that 14-3-3γ was regulated by the UPP.

Jin *et al*. previously reported several putative proteins associated with the 14-3-3γ protein [18]. Among these, USP37 was predicted to be one of the binding partners of 14-3-3γ. On the basis that USP37 might catalyze the deconjugation of polyubiquitin chains from 14-3-3γ, we performed an IP assay with USP37 and 14-3-3γ to validate their interaction. Reciprocal co-IP confirmed that these two proteins interacted with each other, with USP37 strongly binding to 14-3-3γ (Figure 3C). Using an antibody specific for 14-3-3γ, we then analyzed the interaction of endogenous 14-3-3γ with USP37. The results confirmed the interaction between them (Figure 3D). Cell lysates were immunoprecipitated with anti-USP37 and anti-14-3-3γ antibodies to investigate co-binding. As expected, endogenous USP37 and 14-3-3γ bound to each other (Figure 3E). In a GST pull-down assay, Myc-USP37 from cell lysates was pulled-down by GST-14-3-3γ bound beads, suggesting that USP37 directly binds to 14-3-3γ (Figure 3F).

Earlier studies revealed that 14-3-3γ and USP37 were localized in the cytoplasm and nucleoplasm [17, 25]. To obtain additional insights into the localization of 14-3-3γ and USP37, we performed an immunofluorescent experiment to observe their co-localization within cells (Figure 3G). As shown in the left panel of Figure 3G, endogenous 14-3-3γ was localized in both the cytoplasm and the nucleoplasm. However, the localization of 14-3-3γ was altered in the nucleus when USP37 was overexpressed, showing co-localization (Figure 3G, right panel) [24, 25]. As the sequences of 14-3-3 isoforms are similar, we investigated whether the other six known isoforms of 14-3-3 interacted with USP37. The results showed that of these six isoforms, only 14-3-3γ bound to USP37, demonstrating that 14-3-3γ is a unique binding partner of USP37 (Figure 3H, lane 7).

### 14-3-3γ undergoes deubiquitination by the catalytic activity of USP37

**Figure 4:**
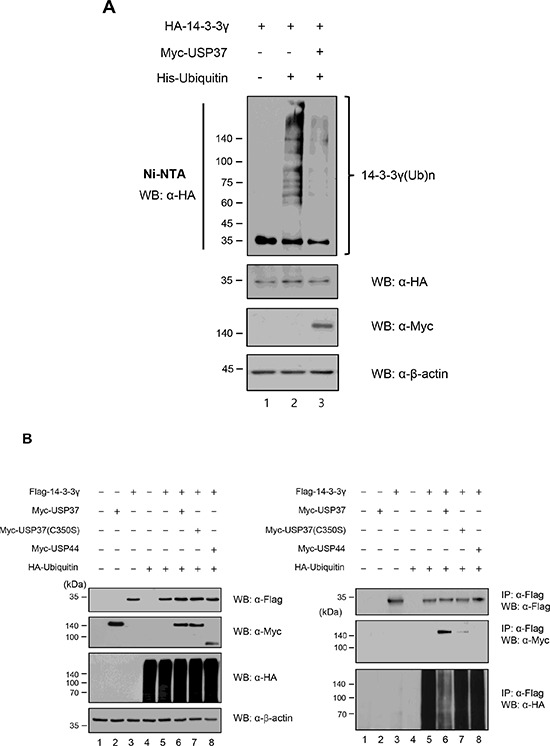

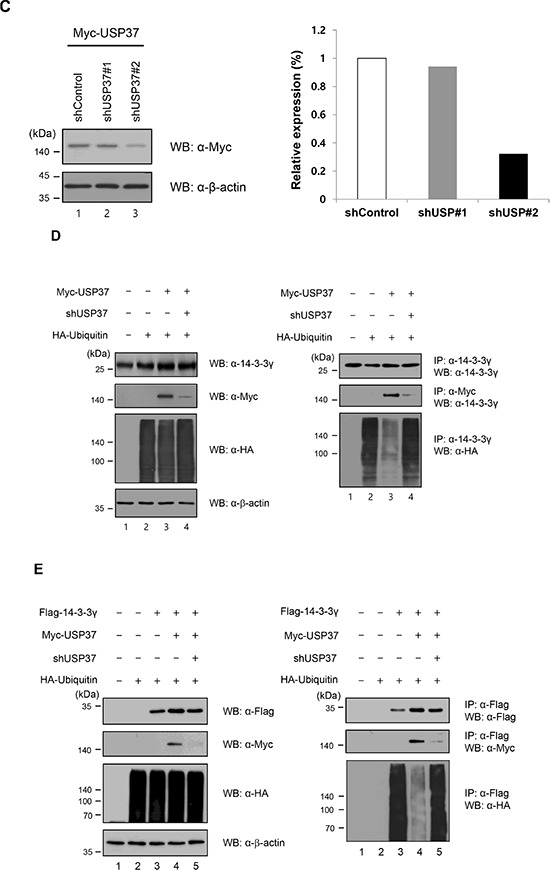

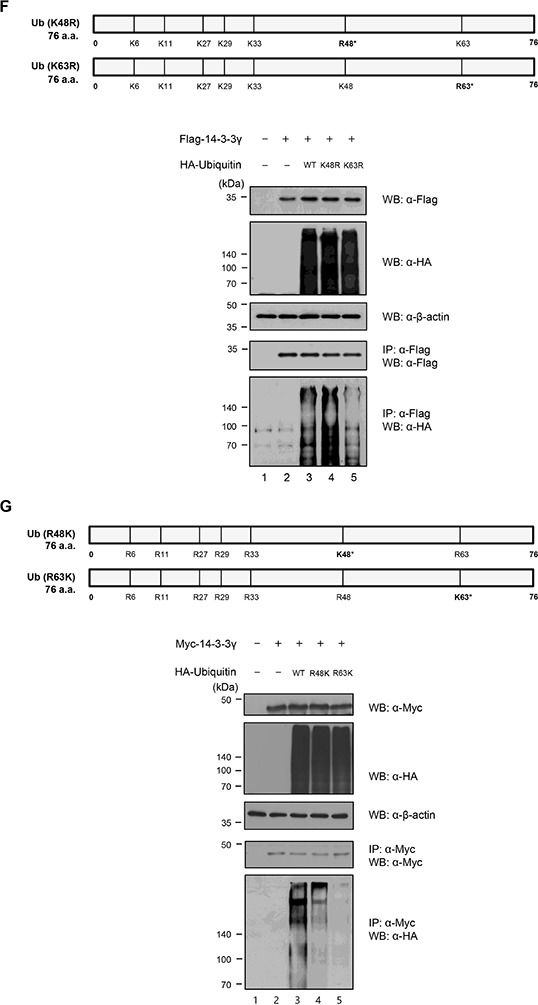

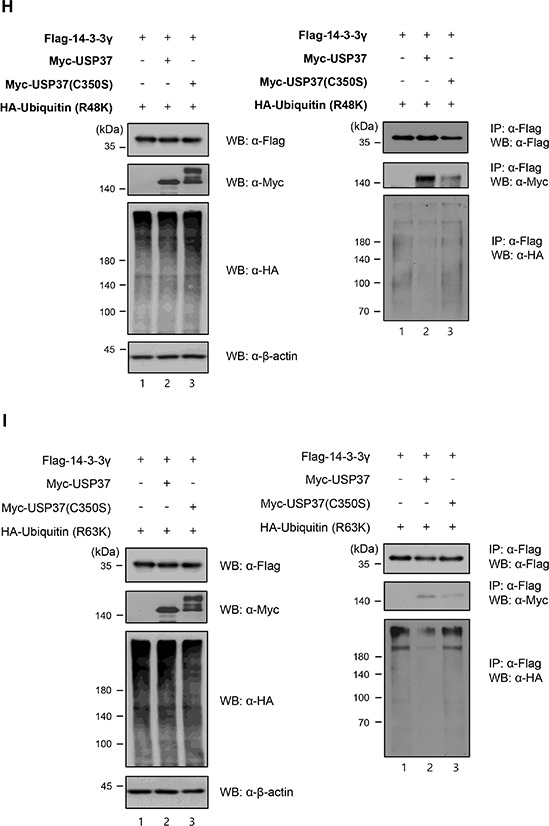
USP37 deubiquitinates 14-3-3γ **A.** HA*-14-3-3γ* was transfected into H1299 cells with or without His*-ubiquitin* and Myc*-Usp37.* The ubiquitination of 14-3-3γ was analyzed using Ni-NTA beads. **B.** Deubiquitination of 14-3-3γ by USP37. 293T cells were transfected with Myc-*14-3-3γ*, HA-*ubiquitin*, and Myc-*Usp37* or with Myc-*Usp37 (C350S)* and Myc-*Usp44*. USP44 was used as a negative control, because it has no DUB activity targeting 14-3-3γ. **C.** Knock-down efficiency specific for USP37. **D.** Deubiquitination of endogenous 14-3-3γ by USP37. 293T cells transfected with HA-*ubiquitin*, Myc-*Usp37*, and sh*Usp37* were immunoprecipitated using an anti-14-3-3γ antibody and blotted with an anti-HA antibody. **E.** 293T cells transfected with HA-*ubiquitin*, Myc-*14-3-3γ*, Myc-*Usp37*, and sh*Usp37* were immunoprecipitated using an anti-14-3-3γ antibody and blotted with an anti-HA antibody to determine the specificity of the DUB activity of USP37 on exogenously ubiquitinated 14-3-3γ. **F.** Lys mutated constructs at Lys-48 and Lys-63. 293T cells were transfected with HA-*ubiquitin (K48R)* or HA-*ubiquitin (K63R)*. **G.** Ubiquitin constructs with alternation of all lysines except Lys-48 or Lys-63, were used in a deubiquitination assay. Cell lysates overexpressing Flag-14-3-3γ, HA-Ubiquitin (R48K), Myc-USP37, and Myc-USP37 (C350S) were used in an IP assay. IP assay was performed with an anti-14-3-3γ antibody. **H.** IP assay was performed with HA-*ubiquitin (R63K)* construct. Myc-USP37 reduced the ubiquitination level of 14-3-3γ, indicating that USP37 specifically regulates Lys-48- and Lys-63-branched ubiquitination.

To determine the functional significance of the interaction between USP37 and 14-3-3γ, we assessed whether the polyubiquitination of 14-3-3γ was deubiquitinated by USP37. For this purpose, cells were transfected with HA-*14-3-3γ*, and His-*Ubiquitin*, with or without Myc-*Usp37* and treated with MG132 before harvesting. Cell lysates were incubated with Ni-NTA beads to refine ubiquitinated proteins. This experiment was performed in urea buffer, which induces protein denaturation. As shown in Figure 4A, polyubiquitination but not monoubiquitination chains of 14-3-3γ precipitated with His-ubiquitin were observed. As expected, the ubiquitination level of 14-3-3γ was decreased by the catalytic activity of Myc-USP37. We then performed a co-IP assay of cell lysates overexpressing Flag-14-3-3γ and HA-ubiquitin, with Myc-USP37, Myc-USP37 (C350S), and USP44. Myc-USP37 (C350S), and USP44 were used as negative controls. Interestingly, the overexpression of USP37 resulted in a significant reduction in the ubiquitination level of 14-3-3γ (Figure 4B, right panel, lane 6). However, USP37 (C350S) and USP44, which were used as negative controls, did not show any DUB enzymatic activity on 14-3-3γ (Figure 4B, lanes 7 and 8). To check the specificity of DUB activity of USP37 on the endogenous 14-3-3γ protein, we designed two kinds of shRNA for *Usp37* and investigated their inhibition of *Usp37* expression (Figure 4C). Using an shRNA #2 with a higher efficiency, we examined the ubiquitination level of endogenous 14-3-3γ. The results revealed a significant increase in the ubiquitination level of endogenous 14-3-3γ in the presence of sh*Usp37* (Figure 4D, lane 4). In contrast, USP37 significantly reduced the ubiquitination level of endogenous 14-3-3γ (Figure 4D, lane 3). Moreover, the specificity of sh*Usp37* was confirmed with exogenous 14-3-3γ (Figure 4E, right panel, lane 5). The formation of the ubiquitination chain depends on specific lysine residues. Ubiquitin-conjugated proteins are destined to undergo degradation via the 26S proteasome and are to have functional activities, including binding and enzymatic activity. To investigate the type of ubiquitination chain generated by 14-3-3γ, we conducted an ubiquitination assay with wild-type ubiquitin and mutant ubiquitins, substituting the Lys-48 or Lys-63 residue with Arg. The aim of this experiment was to investigate and compare whether mutant ubiquitins (K48R, and K63R) were able to generate Lys-48- or Lys-63-branched ubiquitin chains since Lys-63-branched ubiquitin playing a role in various cellular functions instead of protein degradation is becoming important [36]. Figure 4F shows a comparison of the ubiquitination level of 14-3-3γ by the wild-type and the mutants. The ubiquitination level mediated by the mutant ubiquitin (K48R) was higher than that of the wild-type, pointing to the formation of ubiquitin chains with lysine residues other than Lys-48. This is closely connected to the ubiquitin chains including Lys-63, independent degradation by Lys-48. In contrast, the ubiquitination level was decreased when ubiquitin (K63R) was used, suggesting that the degradation of 14-3-3γ was mediated by Lys 48-branched ubiquitination. These results indicate that the ubiquitination of 14-3-3γ was possible through Lys-48 and Lys-63 (Figure 4F). To provide further evidence of the specificity of 14-3-3γ ubiquitination, we performed a co-transfection experiment with HA-*ubiquitin* mutants, substituting Lys with Arg at all positions, except the Lys-48 and Lys-63 positions (Figure 4G). We focused on Lys-48- and Lys-63-branched ubiquitination of 14-3-3γ because USP37 was shown to play a role in Lys-48- and Lys-63-branched deubiquitination [24]. 14-3-3γ was clearly co-precipitated with the HA-ubiquitin mutants, indicating that 14-3-3γ is regulated by both Lys-48- and Lys-63-branched ubiquitination (Figure 4G, lanes 4 and 5). Interestingly, the ubiquitination level of Lys-48 was much higher than that of Lys-63. Taken together, the results suggest that ubiquitination may regulate both the stability and functional effect of 14-3-3γ (Figure 4G). These results led us to investigate whether USP37 regulated Lys-48- and Lys-63-branched polyubiquitination of 14-3-3γ, with regard to deubiquitination. We performed a deubiquitination assay with mutant ubiquitins in cells overexpressing Myc-USP37. As shown in Figure 4H and 4I, USP37, but not USP37 (C350S), clearly reduced the ubiquitination level of 14-3-3γ. Collectively, these findings demonstrate that the ubiquitination system modulates the stability and functional role of 14-3-3γ and that USP37 is a DUB specific for 14-3-3γ.

### USP37 promotes cell proliferation by stabilizing 14-3-3γ

**Figure 5:**
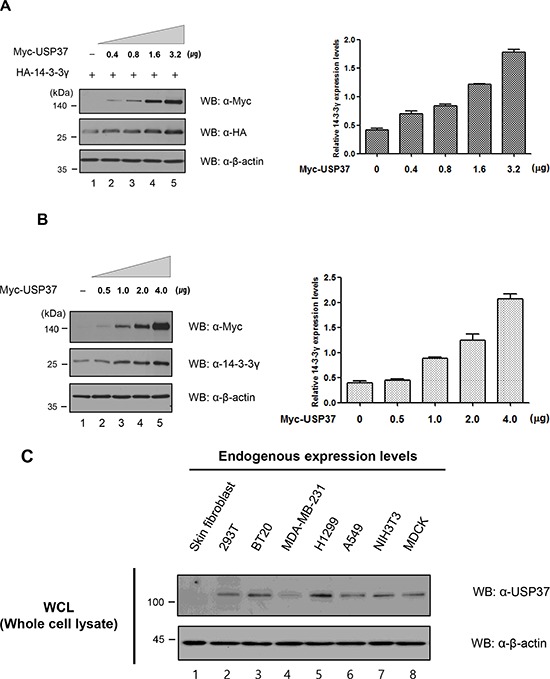

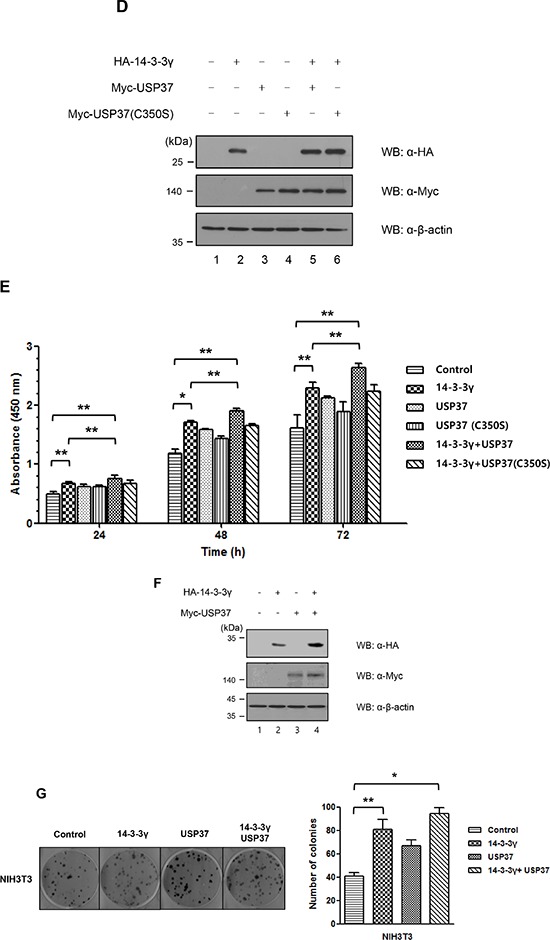

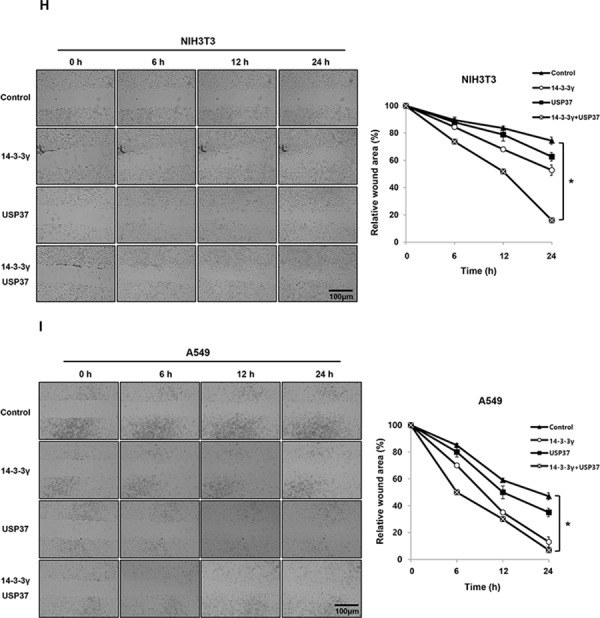
USP37 promoted cell viability and proliferation by stabilizing 14-3-3γ **A.** Exogenous stabilization of 14-3-3γ by USP37. HA-*14-3-3γ* was co-transfected with increasing amounts of Myc-*Usp37* into 293T cells. Western blotting was performed to determine the exogenous level of 14-3-3γ. **B.** Endogenous stabilization of 14-3-3γ by USP37. Myc-*Usp37* only was transfected into 293T cells in a dose-dependent manner. **C.** Various cancer cells and non-transformed human skin fibroblast cells were used to determine the expression level of USP37. **D.** 293T cells were transfected with only Myc-*Usp37* and Myc-*Usp37 (C350S)* together with HA-14-3-3γ to investigate cell viability and proliferation separately. **E.** 293T cells transfected with respective constructs were with a CCK-8 assay. The absorbance value for increased cell viability was graphed using Graph Pad Prism Software. The data are presented as means ± s.d. *P < 0.01 and **P < 0.05, *n* = 3. **F.** NIH3T3 cells were transfected with respective constructs, as described in the binding assay. **G.** After conformation of protein expression, the cells were seeded into 6-well plates and incubated for 14 days. The colonies were stained, and the numbers were determined by counting the colonies. *n* = 3. The results represent the average numbers of colonies that formed in three individual experiments. The numbers of colonies were graphed using Graph Pad Prism Software. The data are presented as means ± s.d. *P < 0.01 and **P < 0.05, *n* = 3. **H and I.** Stably transfected NIH3T3 cells with their respective constructs were used for investigating 14-3-3γ and USP37-mediated migratory and invasive potential in a wound-healing assay. The wound healing by migrated cells at 0, 6, 12 and 24 h was imaged with the JuLI Stage system (NanoEnTek, Pleasanton, CA, USA). The data are representative of four biological replicates. The percentage of the wound area was graphed using Graph Pad Prism Software. The data were presented as means ± s.d. *P < 0.01, *n* = 4. Scale bar = 200 μm.

Based on the finding that 14-3-3γ underwent Lys-48-branched polyubiquitination, we investigated whether the DUB activity of USP37 could stabilize exogenous and endogenous 14-3-3γ. The cells were transfected with a specific amount of HA-*14-3-3γ* only (Figure 5A, lane 1) or co-transfected with increasing amounts of Myc-*Usp37*. USP37 dramatically increased the protein expression level of 14-3-3γ in a dose-dependent manner (Figure 5A, lanes 2–5). The endogenous level was confirmed through dose-dependent transfection experiments with Myc-*Usp37* (Figure 5B). USP37 also increased the endogenous level of 14-3-3γ in a dose-dependent manner, as shown by the exogenous level. These results indicate that the DUB activity of USP37 inhibits the degradation pathway of Lys-48-branched polyubiquitinated 14-3-3γ.

### USP37 contributes to cancer progression, together with 14-3-3γ

Previous studies showed that USP37 is an oncogenic protein [25–27]. Thus, we estimated the expression level of USP37 in various cancer cells and in normal cells, such as human skin fibroblasts (Figure 5C). We found that the expression level of USP37 was higher in cancer cells. Several recent studies and our previous study demonstrated that 14-3-3γ was a key regulator of cell proliferation and that it played a critical role in cell growth, survival, and tumorigenesis [15, 16]. The identification of the interaction between USP37 and 14-3-3γ led us to investigate whether USP37 was associated with cell proliferation and migration via the regulation of 14-3-3γ. We transfected 293T cells with HA-*14-3-3γ* alone or co-transfected the same cells with Myc-*Usp37* and Myc-*Usp37 (C350S)* (Figure 5D), and performed a cell proliferation assay (Figure 5E). We used a cell counting kit-8 (CCK-8) to measure the cell viability and proliferation mediated by the interaction between HA-14-3-3γ and Myc-USP37. The cell viability of the cells transfected with HA-*14-3-3γ* and Myc-*Usp37* was increased in each cell line at 24 h, 48 h, and 72 h, as measured by absorbance at 450 nm. The cell viability of the transfected cells with HA-*14-3-3γ* and Myc-*Usp37* was increased compared with that of a mock control. Importantly, the cell viability of the co-transfected cells was clearly increased compared with that of the Myc-*Usp37*, Myc-*Usp37 (C350S)*, or HA-*14-3-3γ* transfected cells (Figure 5E). Overexpression of USP37 and 14-3-3γ resulted in the generation of the highest number of colonies compared with other test conditions, indicating that USP37 is involved in proliferation (Figure 5F and 5G). At the same time, we carried out a wound-healing assay with the same NIH3T3 cells as used in the colony-forming unit (CFU) experiment. Interestingly, the wound area was reduced up to 80% in the cells overexpressing USP37 and 14-3-3γ (Figure 5H). Similar results were obtained with A549 cells (Figure 5I).

**Figure 6:**
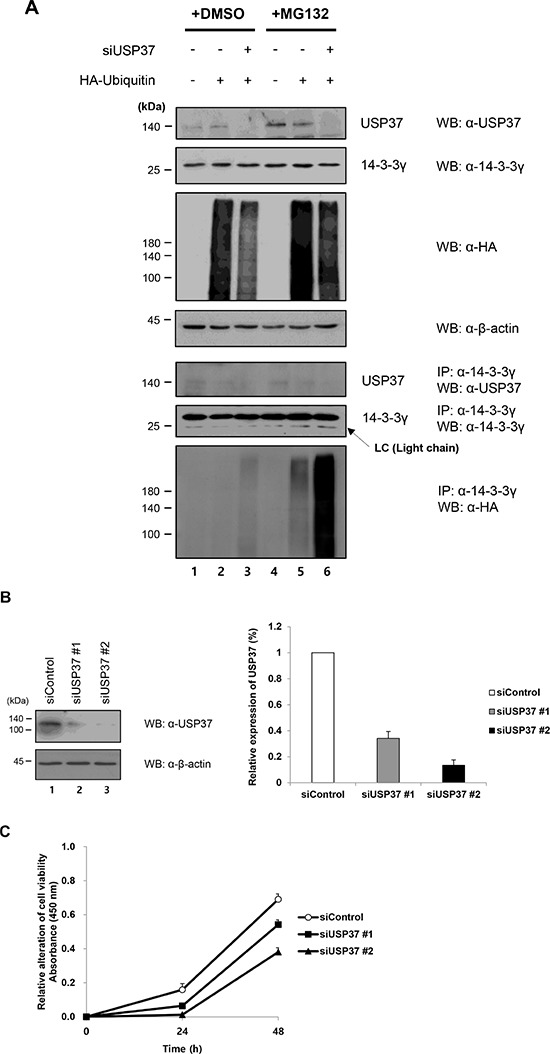

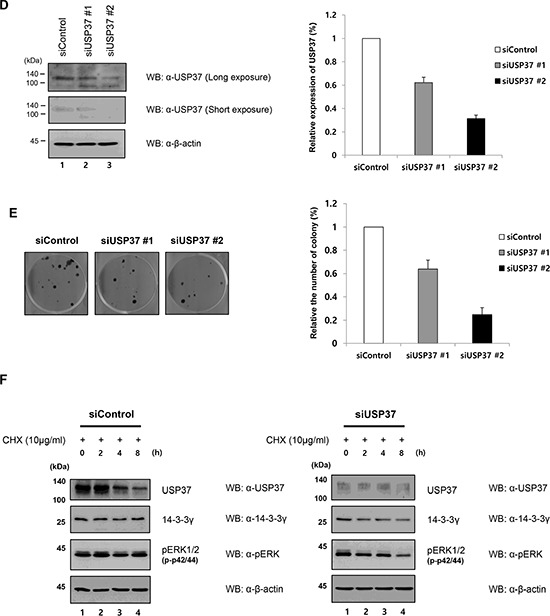

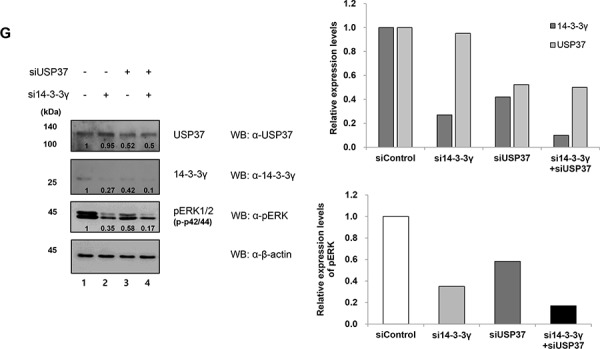
Knock-down effect of USP37 in cancer cells **A.** H1299 cells were transfected with HA-*ubiquitin* and si*Usp37*, and then treated with MG132. Harvested cells were immunoprecipitated with an anti-14-3-3γ antibody and proteins were detected with indicated antibodies. **B and C.** Two kinds of siRNA specific for USP37 induced a knock-down effect and these cells were used to investigate the cell viability. The results represent the average expression of USP37 and relative cell viability from three independent experiments. The data are presented as means ± s.d. *n* = 3. **D and E.** A CFU assay was performed, and we carried out transfection with si*Usp37* #1 and si*Usp37* #2. The same number of these cells was seeded into 6-well plates. The results represent the average expression of USP37 and the average number of cell viability from three independent experiments. The data are presented as means ± s.d. *n* = 3. **F.** To compare the half-life of 14-3-3γ under USP37-depleted and USP37-undepleted conditions, we determined time-dependent changes in the levels of 14-3-3γ protein. These cells were incubated in medium containing CHX (100 μM). In addition, we investigated the level of phosphorylated ERK to determine whether USP37 was directly associated with cell proliferation, together with 14-3-3γ. The results represent the average expression of 14-3-3γ following knock-down of USP37 from three independent experiments. The data are presented as means ± s.d. *n* = 3. **G.** A549 cells transfected with their respective siRNAs were used for checking the knock-down effect on MAPK signaling pathway. The relative expression level of USP37, 14-3-3γ, and pERK was estimated using Image J software program (NIH, Bethesda, MD, USA).

A recent study suggested that knock-down of specific DUBs was required to investigate their roles in regulating target proteins [28]. To monitor the ubiquitination level of 14-3-3γ, HA-*ubiquitin* was transfected into 293T cells, in which the expression of USP37 was downregulated. (Figure 6A). In contrast to the overexpression, the depletion of USP37 had an opposite effect on 14-3-3γ, resulting in the upregulation of ubiquitin conjugation. When the cells were treated with MG132, higher quantities of ubiquitinated 14-3-3γ accumulated in the USP37-depleted cells (Figure 6A, lanes 5 and 6). Figure 5 shows the results of a cell proliferation assay performed in USP37-depleted cells. First, we measured the cell viability 0, 24, and 48 h after seeding. The depletion of USP37 reduced the cell viability compared with a control (Figure 6B and 6C). In a CFU assay, colony formation declined in the USP37-depleted cells, as occurred when 14-3-3γ was depleted (Figure 6D and 6E). Based on the efficiency of knock-down for USP37 expression, we used si*Usp37* #2 to determine the half-life of 14-3-3γ. Depleted and non-depleted cells were treated with cycloheximide (CHX) and harvested in a time-course manner (Figure 6F). Interestingly, the stability of 14-3-3γ dramatically was declined in the cells expressing a low level of USP37. The difference in the level of 14-3-3γ in the presence of USP37 suggested that USP37 regulated the stability of 14-3-3γ. In addition, we monitored phosphorylation of ERK (p42/44), which is one of downstream components of MAPK signaling in cells expressing USP37. The levels of pERK gradually decreased over time in USP37-depleted cells in contrast to those in USP37-nondepleted cells. To provide further evidence that USP37 regulates the stability of 14-3-3γ, we carried out co-knock-down experiment with si*14-3-3γ* and si*Usp37*. The endogenous level of 14-3-3γ and pERK were declined by si*Usp37* compared with a control (Figure 6G). It is of interest that the lowest level of pERK was shown in the cells, which 14-3-3γ and USP37 were knocked down (Figure 6G, lane 4). These findings indicate that USP37 participates in the regulation of cell proliferation, and that USP37 induces stability of 14-3-3γ. Taken all together, USP37 has tumorigenic potential following MAPK pathway by stabilization of 14-3-3γ and plays an important role in cancer progression.

These findings indicate that USP37 participates in the regulation of cell proliferation, and that USP37 induces stability of 14-3-3γ. They play an important role in cancer progression.

## DISCUSSION

Isoforms of 14-3-3 share 70–80% amino acid identity. They are a ubiquitous family of molecules and participate in the protein kinase signaling pathways of all eukaryotic cells. Functioning as a phosphoserine/phosphothreonine-binding molecule, 14-3-3 participates in phosphorylation-dependent protein-protein interactions, cell cycle progression, initiation and maintenance of DNA damage checkpoints, apoptosis, cell proliferation, and cell survival [18, 22, 29–31].

Many studies have demonstrated that 14-3-3γ promoted cell proliferation by activating MAPK and PI3K signaling pathways and that 14-3-3γ caused mitotic checkpoint defects in a human lung cancer cell line, resulting in abnormal DNA replication and polyploidization [15, 17, 32]. We previously showed that 14-3-3γ played a pivotal role in the cell proliferation and survival signaling pathways of fibroblastic cells [16]. Mass spectrometry analysis revealed that 14-3-3γ was associated with several proteins that function in cellular communication, signal transduction, protein synthesis, and cellular organization. Among these proteins, USP37 was predicted to be a putative binding candidate of 14-3-3γ [18, 33, 34].

It was reported that USP37 induced the stability of the oncogenic fusion protein PLZF/RARA. PLZF/RARA acts as an oncogenic transcriptional regulator in leukemia and as an important regulator in cell cycle progression, which is induced by diverse checkpoint regulators [27]. USP37 was shown to induce the stability of cyclin A by deubiquitination, resulting in an accumulation of cyclin A, which leads to the G1-S phase transition [35]. USP37 was also shown to regulate cell proliferation and the Warburg effect by stabilizing the level of c-Myc [26].

In *de novo* studies of a mouse model in which Ba/F3 cells were stably expressed with 14-3-3γ, tumors of various sizes developed at the transplanted sites on mice flanks, illustrating the oncogenic potential of Ba/F3 cells. In contrast, no tumors developed in mock-transfected Ba/F3 cells (Figure 1A and 1B). The subsequent histochemical analysis of the excised mouse tumors revealed the augmentation of cell proliferative markers, such as c-Myc and PCNA, with oncogenic potential (Figure 1C and 1D). In addition, we compared the endogenous expression levels of USP37 and 14-3-3γ in various cancer cell lines with those in normal cells and found lower expression levels of these proteins in the normal cells (Figure 2A). In the present study, we showed that 14-3-3γ mediated cell proliferation in MCF7, A549, H1299, and NIH3T3, leading to neoplastic transformation and anchorage-independent growth *in vitro* (Figure 2A–2G). The findings of our study and those of previous studies suggest that 14-3-3γ plays a role in the development and progression of cancer [15, 16, 22, 30, 32].

As USP37 is a known putative substrate for 14-3-3γ [18], it was overexpressed in cells to determine its interaction with exogenous and endogenous 14-3-3γ (Figure 3C–3E). A recent study demonstrated that HBx, an oncoprotein of the hepatitis B virus and a regulator of hepatocarcinogenesis, promoted the translocation of USP37 from the nucleoplasm to the cytoplasm and that the degradation of USP37 seemed to be prevented by the E3 ligases, APC/CDH1 and SCF/β-TrCP [25]. In the present study, we confirmed that 14-3-3γ co-localized with USP37 in the nucleoplasm and cytoplasm in an immunofluorescent assay, and we showed that it directly bound to USP37 in a GST pull-down assay (Figure 3F and 3G). To shed light on the ubiquitination of mammalian 14-3-3γ, we performed an ubiquitination assay. We demonstrated that 14-3-3γ was polyubiquitinated and that its ubiquitination increased in the presence of a proteasomal inhibitor, MG132 (Figure 3A and 3B).

Specific DUBs for 14-3-3γ have not been identified. In the present study, we investigated whether USP37 regulated the ubiquitination of 14-3-3γ because the enzymatic activity of USP37 has already been reported to induce the stabilization of target proteins [25, 26]. In this study, we observed that the ubiquitination level of 14-3-3γ decreased when USP37 was overexpressed. However, it did not decline with a USP37 (C350S) mutant, sh*Usp37*, and USP44 which were used for negative controls (Figure 4B, 4D, and 4E). In the majority of cases, cellular proteins undergo ubiquitination through seven lysine residues [36]. We carried out Lys-48- and Lys-63-branched ubiquitination and deubiquitination assays to identify the ubiquitination chain of the target protein (Figure 4F and 4G). Consistent with our results, recent studies also showed that USP37 was involved in Lys-48- and Lys-63-branched deubiquitination [24, 35] (Figure 4H and 4I). We confirmed that 14-3-3γ underwent Lys-specific ubiquitination, showing that ubiquitin played a key role in various functions as one of scaffolding proteins and in degradation. As described above, our results suggest that the enzymatic activity of USP37 targets 14-3-3γ with specificity for Lys-48- and Lys-63-branched ubiquitination.

Recent reports demonstrated that USP37 had diverse regulatory functions in the cellular signaling pathway and that these played a role in malignancy, including promoting the proliferation and viability of cancer cells [24–27, 35]. To shed additional light on the role of USP37, we conducted additional functional studies, including the measurement of cell proliferation and the determination of whether USP37 was involved in the regulation of 14-3-3γ. We showed that USP37 was a specific DUB, which prevented the degradation of 14-3-3γ. It might be helpful in further investigations of the regulation of 14-3-3γ-mediated signal transduction, as it relates to cell proliferation. We previously performed wound-healing and CFU assays and demonstrated that 14-3-3γ promoted cell proliferation and migration. In the present study, we performed additional assays to investigate whether cell proliferation increased when USP37 was overexpressed (Figure 5). Notably, our results showed that overexpression of USP37, but not USP37 (C350S), enhanced the proliferation of cancer cells when 14-3-3γ was co-transfected (Figure 5D–5I). Recent studies reported that USP37 was associated with the regulation of cell proliferation in cancers [25–27]. Thus, we examined whether knock-down of USP37 decreased cancer cell growth by downregulation of 14-3-3γ (Figure 6). We confirmed the stability of 14-3-3γ and cell proliferation in the USP37-depleted cells, suggesting that USP37 affected functions involved in 14-3-3γ. A rapid decrease in 14-3-3γ levels could explain the decreased pERK levels in USP37-depleted cells. As shown in Figure 6G, the cells expressing the low level of 14-3-3γ and USP37 showed more decreased level of pERK than that in only 14-3-3γ-depleted cells, suggesting that USP37 is involved in MAPK signaling pathway. Our results demonstrate that knock-down of USP37 plays a critical role in the progression of cancer by decreasing the ubiquitination and stability of 14-3-3γ.

The findings on the regulation of 14-3-3γ ubiquitination by USP37 establish a new role for USP37 in cancer cell proliferation, indicating that ubiquitination, a PTM, has an important role in cancer. As USP37 is a specific DUB for 14-3-3γ, it might provide an effective and selective target for novel cancer therapies.

## MATERIALS AND METHODS

### Tumorigenicity assay

Stably expressing Ba/F3 cells with mock and Myc-*14-3-3γ* were used for the assay of tumorigenicity. 2 × 10^6^ cells/ml were injected subcutaneously into < 12 weeks old young (NOD.CB17-Prkdc SCID/J) from the Jackson's laboratory in the United States. Mice were observed for 6 weeks for signs of palpable or visible tumors at the site of injection. Tumorigenic cell lines gave rise to visible pea-sized mass, which represented malignant tumors, and in non-tumorigenic cell lines no evidence of tumors for up to 3 months after injection was shown. Tumor volumes were graphed using Graph Pad Prism Software (GraphPad Software, 7825 Fay Avenue, Suite 230 La Jolla, CA, USA).

### Cell lines, cell cultures, and transfection

Ba/F3 cells were maintained in RPMI 1640 medium supplemented with 10% fetal bovine serum (FBS) and 10 pM IL-3 (PeproTech, London, UK). Transfection of Ba/F3 cells were carried out using Bio-Rad Gene Pulsar II (Hercules, CA, USA) set at 300 V, 960 μF [37]. 293T, MCF-7, A549, H1299, and NIH3T3 cells were grown in DMEM supplemented with 10% FBS and, 1% penicillin and streptomycin (Gibco-BRL, Rockville, MD, USA). To observe expression and knock-down of genes, transfection was carried out using polyethyleneimine (PEI) (Polysciences, Warrington, PA, USA) and Lipofectamine 2000 (Invitrogen, Paisly, UK). Human skin fibroblast cells were used to compare the endogenous expression level of USP37 and 14-3-3γ.

### Expression constructs and antibodies

Full-length cDNAs for murine 14-3-3 isoforms (Genbank: AF058799.1, U57312.1, U57311.1, U79231.1, AF058798.1, AF058797.1, and Z19599.1) and human USP37 (Genbank: NM_020935.2) were cloned into the Flag, Myc, HA and GST epitope encoded vectors, respectively. A full-length cDNA for *Usp37* was purchased (Onegene Bio, Seongnam, Korea). Mutant ubiquitin constructs used for ubiquitination assay were generated as previously described [38]. Antibodies for anti-14-3-3γ, anti-GST, anti-β-actin, anti-Myc, anti-HA (Santa Cruz Biotechnology, Santa Cruz, CA, USA), anti-USP37 (Bethyl Laboratories, Montgomery, USA), and anti-pERK (phospho-p42/44) (Cell Signaling Technology, Beverly, MA, USA) were used for Western blotting and IP.

### Knock-down by RNA interference

siRNA for 14-3-3γ was generated with following sequence: #1 (5′-GCT ACT ACT GCA GTC TTT A-3′), #2 (5′-AGG GTC ATC AGT AGC ATT GA-3′). Two kinds of shRNAs for USP37 were constructed (Onegene Bio, Seongnam, Korea) using pSilencer 1.0-U6 system (Ambion, Austin, TX, USA). And two kinds of siRNAs were used for knock-down of USP37. The mRNA target sequences chosen for designing following: #1 (5′-CTT GGA AGA CTG AAC CTG T-3′), and #2 (5′-GAU UUG ACA GAA UGA GCG A-3′).

### Site-directed mutagenesis

The *Usp37 (C350S)* mutant was generated using a QuikChange™ site-directed mutagenesis kit (Stratagene, La Jolla, CA, USA) according to the manufacturer's instructions. In the PCR step, the forward primer (5′-T TTG GGA AAT ACC AGC TAT ATG AAT GC-3′) and reverse primer (5′-GCA TTC ATA TAG CTG GTA TTT CCC AAA-3′) were used for replacing a cysteine with a serine at position 350 of USP37.

### Immunohistochemistry

Tumor tissues were fixed in 4% paraformaldehyde within PBS at 4°C overnight, embedded in paraffin, and sectioned at a thickness of 5 mm. The sections were deparaffinized with xylene, and rehydrated in graded ethanol. Endogenous peroxidase activity was blocked with 3% hydrogen peroxide in methanol at room temperature for 10 min. In antigen retrieval, a heat-induced procedure was performed using a microwave at 95°C to 100°C for 15 min, in a 0.01 M citrate acid buffer (pH 6.0). After non-specific binding was blocked by 10% normal goat serum in PBS for 30 min at room temperature, the sections were incubated overnight at 4°C with a primary antibody, rabbit polyclonal anti-14-3-3γ (Santa Cruz Biotechnology, Santa Cruz, CA, USA). The sections were rinsed with PBS and incubated with streptavidin-biotin-peroxidase complex secondary antibody goat anti-rabbit IgG (Santa Cruz Biotechnology, Santa Cruz, CA, USA) for 30 min at room temperature. The slides were incubated with 3,3′-diaminobenzidine chromogen for 5 to 10 min at room temperature and washed with distilled water. Finally, sections were counterstained with hematoxylin for 2 min followed by dehydration and mounting.

### Immunofluorescent assay

After transfection, NIH3T3 and A549 cells were seeded (3 × 10^3^ per 12 mm flame sterilized coverslips) and were used for immunofluorescent assay performed as previously described [28].

### Stabilization assay

293T cells were co-transfected with a constant amount of Flag-*14-3-3γ* (0.5 μg) together with or without increasing amount of Myc-*Usp37* (0, 0.4, 0.8, 1.6, and 3.2 μg). For checking the level of endogenous 14-3-3γ, cells were transfected with Myc-*Usp37* (0, 0.5, 1.0, 2.0, 4.0 μg). After 24 h of transfection, these cells were harvested and subjected to immunoblotting with the indicated antibodies.

### Cycloheximide (CHX) chase assay

For blocking further protein synthesis of 14-3-3γ, indicated cells were incubated in DMEM containing 100 μM CHX (Sigma-Aldrich, St. Louis, MO, USA). These cells were harvested for 8 h consecutive time points after CHX treatment and cells lysates were analyzed by immunoblotting.

### His-ubiquitin pull-down assay

293T cells transfected with HA-*14-3-3γ*, Myc-*Usp37,* and His-*ubiquitin* for 24 h were harvested and re-suspended in urea buffer (pH 8.0). The ingredient of urea buffer is illustrated in a previous study [39]. The lysates were incubated with Ni-NTA beads (Merck KGaA, Darmstadt, Germany) for 4 h at room temperature. The beads were washed with urea buffer B (pH 6.3) and washing buffer.

### GST pull-down assay

14-3-3γ subcloned into glutathione S-transferase (GST)-tagged vector was transformed and expressed in BL21 *E. coli* strain. Bacterial lysate expressing GST-14-3-3γ was purified using Glutathione-Sepharose Beads (GE Healthcare, Buckinghamshire, England), according to the manufacturer's instructions. Purified GST-14-3-3γ proteins were confirmed by Coomassie Brilliant Blue staining. To check the interaction between these proteins, GST or GST-14-3-3γ combined beads were incubated with 293T cell lysates overexpressing Myc-USP37 and were tested by immunoblotting using anti-GST and anti-Myc antibodies.

### Western blotting and IP (IP) assay

For all the experiments, the indicated cells were subsequently lysed in an ice-cold lysis buffer and were used for Western blotting as previously described [28]. For co-IP assay, cell lysates were incubated with antibodies at 4°C overnight. Protein-A/G beads were then added and rotated, using a rotor at 4°C for 1 h. For the *in vivo* ubiquitination and deubiquitination assays, cells were lysed and immunoprecipitated in denaturation conditions. We detected with antibodies to check precipitation for ubiquitination of target proteins.

### Cell proliferation by cell counting and colony formation assays

After 24 h of transfection of plasmid DNAs and siRNAs, each of transfected cells was plated with the same number in 24-well culture dish. The cell counting was performed. O.D. was measured at 450 nm to determine the cell viability in each well by using cell counting kit-8 (CCK-8) (Dojindo Molecular Technologies, Rockville, MD, USA). For colony formation assay, the colonies were fixed in ice cold methanol for 10 min, and stained with 1% crystal violet in methanol for 15 min. The colonies were finally rinsed with PBS, and then counted.

### Wound healing assays

Indicated cells were seeded in 6-well plates and wounded by manual scratching with 200 μl pipette tips, washed with PBS, and incubated at 37°C in complete media. At the indicated time points, phase contrast images at specific wound sites were captured at 0, 12, 24 and 36 h. The original and recovered wound areas of these cells were determined by Image J software program (NIH, Bethesda, MD, USA). The percentage of migration was measured as the recovered wound area relative to the original wound area. Tumor volumes were graphed using Graph Pad Prism program (Graph Pad Prism Software, San Diego, CA, USA). In addition, we captured wound areas in indicated cells at 0, 12, and 24 h using JuLI Stage, a live cell movie analyzer (NanoEnTek, Pleasanton, CA, USA).
